# Supplementary figures and images for: Apoptosis is not conserved in plants as revealed by critical examination of a model for plant apoptosis-like cell death
Source: BMC Biol. 2021 May 12;19:100. doi: 10.1186/s12915-021-01018-z (PMC8117276; doi:10.1186/s12915-021-01018-z)

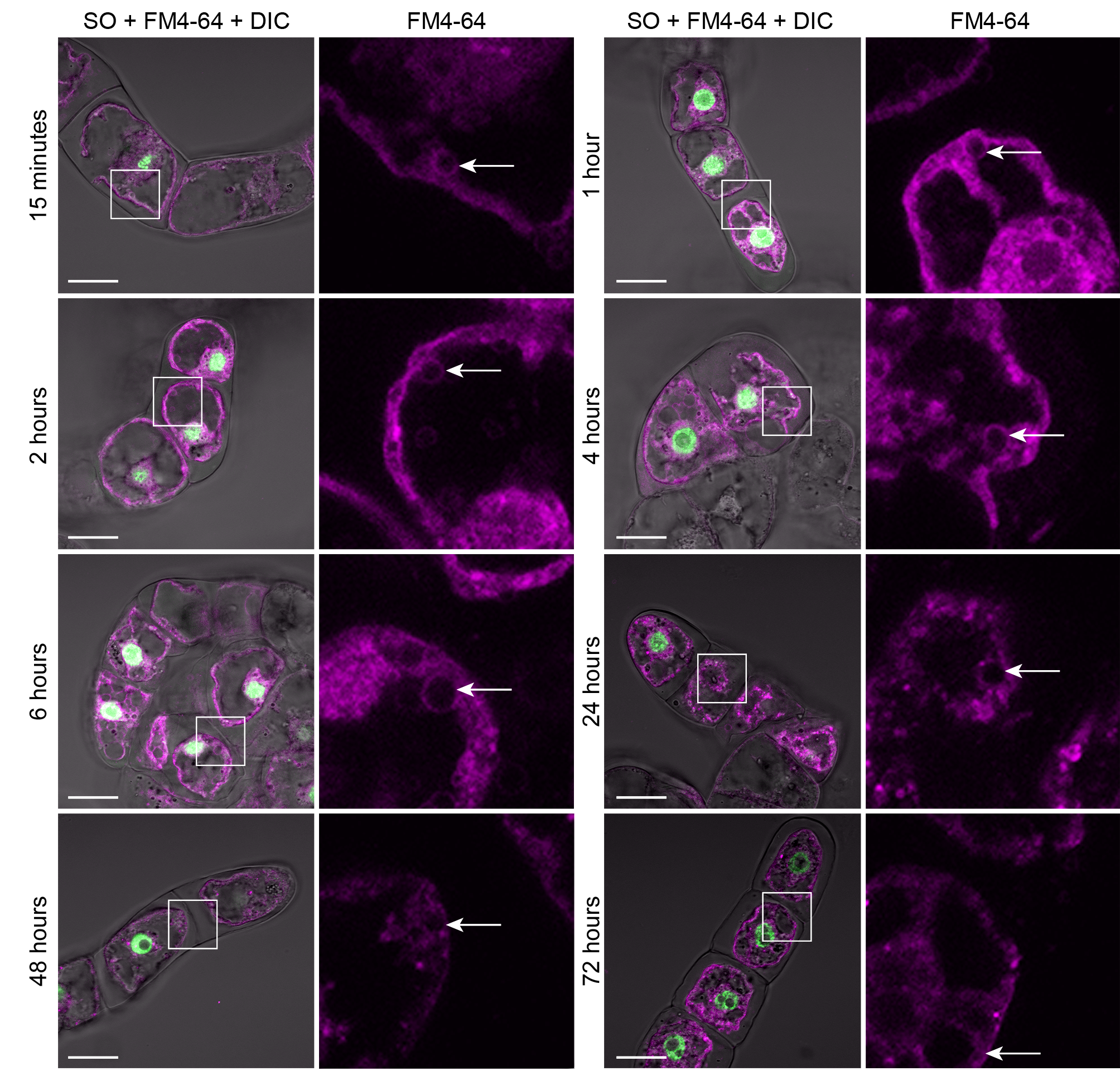

Supplement: Supplementary file 2 — Additional file 2: Figure S1. Time course analysis of gross morphological changes following 55 °C HS. FM4–64 and SO dyes were used to visualize collapsed cells with permeabilized PM. After a 10-min HS at 55 °C, BY-2 cells were stained and imaged at several time points ranging from 15 min to 72 h. Staining was performed as described in protocol iv (see Methods, Sytox Orange and FM4-64 staining). FM4–64 panels represent the corresponding boxed areas. Arrows indicate FM4–64 positive vesicles that were found within the boundaries of cell corpses. DIC, differential interference contrast. Scale bars, 20 μm. Figure S2. HS-induced cell death is ATP- and Ca2+-independent process. Morphology of FDA-stained cells under normal conditions (No HS) and after a 55 °C HS. Protoplast shrinkage is denoted by arrows. Pre-treatment with 48 μM CCCP for 10 min, 15 μM CsA for 2 h, or 10 mM EGTA for 10 min did not alleviate protoplast shrinkage upon HS. Each treatment was repeated at least twice. Scale bars, 20 μm. [file 12915_2021_1018_MOESM2_ESM.zip › Additional file 2_FigS1.tif]

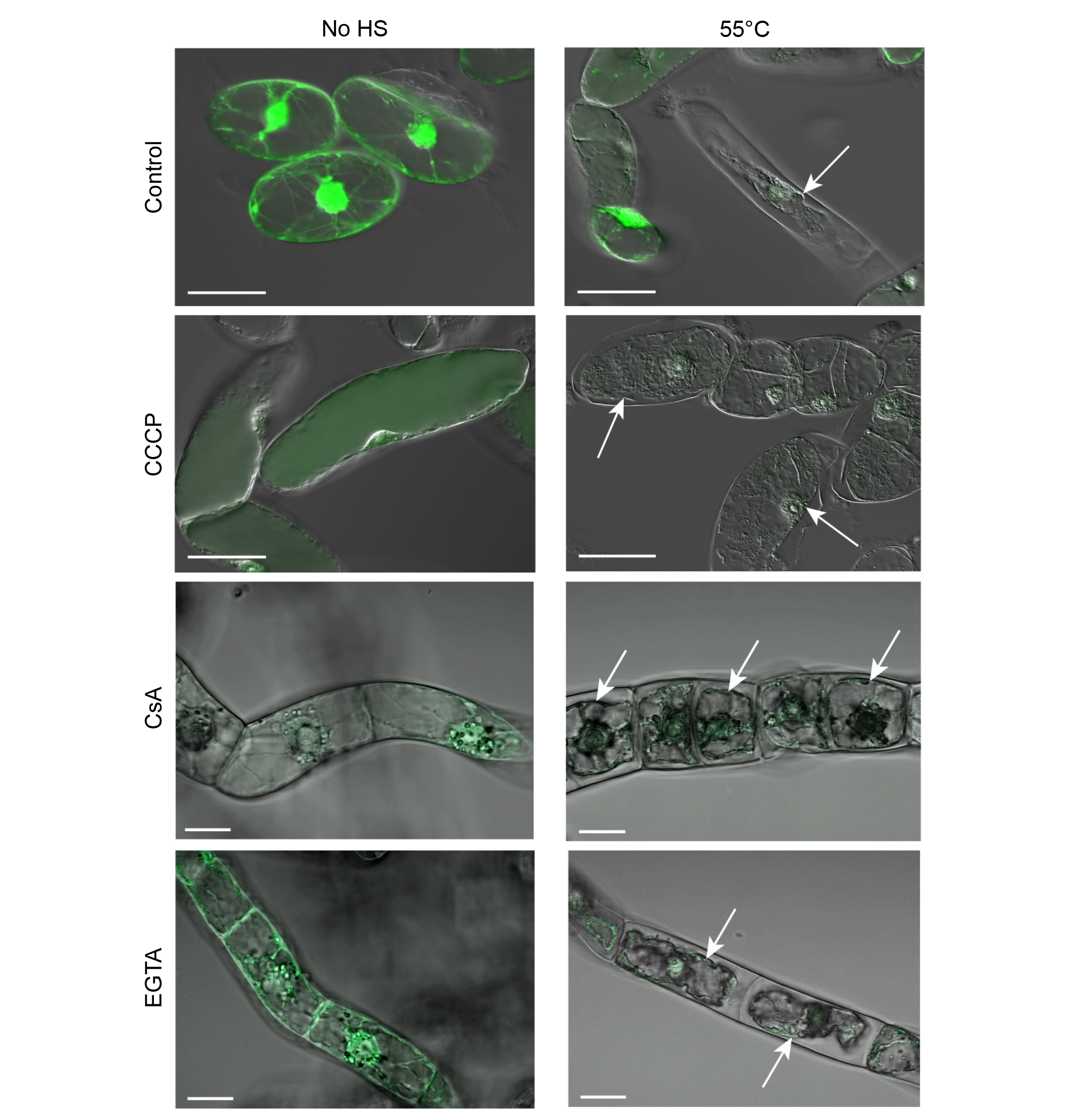

Supplement: Supplementary file 2 — Additional file 2: Figure S1. Time course analysis of gross morphological changes following 55 °C HS. FM4–64 and SO dyes were used to visualize collapsed cells with permeabilized PM. After a 10-min HS at 55 °C, BY-2 cells were stained and imaged at several time points ranging from 15 min to 72 h. Staining was performed as described in protocol iv (see Methods, Sytox Orange and FM4-64 staining). FM4–64 panels represent the corresponding boxed areas. Arrows indicate FM4–64 positive vesicles that were found within the boundaries of cell corpses. DIC, differential interference contrast. Scale bars, 20 μm. Figure S2. HS-induced cell death is ATP- and Ca2+-independent process. Morphology of FDA-stained cells under normal conditions (No HS) and after a 55 °C HS. Protoplast shrinkage is denoted by arrows. Pre-treatment with 48 μM CCCP for 10 min, 15 μM CsA for 2 h, or 10 mM EGTA for 10 min did not alleviate protoplast shrinkage upon HS. Each treatment was repeated at least twice. Scale bars, 20 μm. [file 12915_2021_1018_MOESM2_ESM.zip › Additional file 2_FigS2.tif]
